# Best practice guidelines for the diagnosis, evaluation, and management of cognitive disorders in Parkinson’s disease

**DOI:** 10.1093/ageing/afag063

**Published:** 2026-03-23

**Authors:** Dana Pourzinal, Deborah Brooks, Deepa Sriram, Emily Mccann, James M King, Nancy A Pachana, Kirstine Shrubsole, Brian Wood, Alexander Lehn, Rodney Marsh, Jacki Liddle, Leander K Mitchell, John D O'Sullivan, Edwin C K Tan, Neil Page, Elton H Lobo, Martie-Louise Verreynne, Sabrina Lenzen, Nadeeka Dissanayaka

**Affiliations:** The University of Queensland, UQ Centre for Clinical Research, Brisbane, Queensland, Australia; The University of Queensland, UQ Centre for Clinical Research, Brisbane, Queensland, Australia; The University of Queensland, UQ Centre for Clinical Research, Brisbane, Queensland, Australia; The University of Queensland, UQ Centre for Clinical Research, Brisbane, Queensland, Australia; The University of Queensland, UQ Centre for Clinical Research, Brisbane, Queensland, Australia; The University of Queensland, School of Psychology, Brisbane, Queensland, Australia; The University of Queensland, Queensland Aphasia Research Centre, Brisbane, Queensland, Australia; The University of Queensland and Metro North Health, STARS Education and Research Alliance, Surgical Treatment and Rehabilitation Service (STARS), Brisbane, Queensland, Australia; The University of Queensland, School of Health and Rehabilitation Sciences, Brisbane, Queensland, Australia; Redland Hospital, Metro South Movement Disorders Service, Cleveland, Queensland, Australia; Princess Alexandra Hospital, Department of Neurology, Woolloongabba, Queensland, Australia; Queensland University of Technology, School of Biomedical Sciences, Brisbane, Queensland, Australia; The University of Queensland, UQ Centre for Clinical Research, Brisbane, Queensland, Australia; The University of Queensland, School of Health and Rehabilitation Sciences, Brisbane, Queensland, Australia; The University of Queensland, School of Psychology, Brisbane, Queensland, Australia; The University of Queensland, UQ Centre for Clinical Research, Brisbane, Queensland, Australia; Royal Brisbane and Women's Hospital, Department of Neurology, Herston, Queensland, Australia; The University of Sydney Faculty of Medicine and Health, School of Pharmacy, Sydney, New South Wales, Australia; The University of Queensland, UQ Centre for Clinical Research, Brisbane, Queensland, Australia; The University of Queensland, UQ Centre for Clinical Research, Brisbane, Queensland, Australia; The University of Queensland, Business School, Brisbane, Queensland, Australia; The University of Queensland, Centre for the Business and Economics of Health, Brisbane, Queensland, Australia; The University of Queensland, UQ Centre for Clinical Research, Brisbane, Queensland, Australia

**Keywords:** cognitive impairment, Parkinson’s disease, dementia, guidelines, older people

## Abstract

Although cognitive impairment is prevalent in people living with Parkinson’s disease (PD), the clinical approach to cognitive disorders in PD varies significantly across health services. Here, we present updated best practice guidelines to standardise the diagnosis, evaluation, and management of cognitive disorders in PD across clinical contexts. Guideline development followed a two-phase process incorporating both expert and lived-experience perspectives. In Phase 1, preparatory research (literature reviews and a national survey) generated 58 preliminary recommendations. These were refined through a modified Delphi process with 29 clinician and research experts, resulting in 51 evidence-based and expert-endorsed recommendations. In Phase 2, perspectives of people with lived experience of cognitive disorders in PD (*n* = 15) were attained through focus groups, which produced 25 recommendations. A subsequent national survey (*n* = 81) demonstrated consensus on 24 of the 25 recommendations. Overall, the guideline development process yielded 58 unique recommendations, including recommendations for a tailored neuropsychological toolkit sensitive to cognitive decline in PD. These are the first best practice guidelines for the diagnosis, evaluation and management of cognitive disorders in PD informed by empirical evidence, expert consensus, and insights from people with lived experience. Clinical adoption of these guidelines will improve the quality of care, diagnostic accuracy, and early detection of cognitive disorders in PD. Future service models should consider incorporating these guidelines to optimise cognitive care in PD and promote evidence-based and patient-centred standards of practice.

## Key Points

- Lack of consistent approaches to managing cognitive disorders in Parkinson’s affects diagnosis and care.
- Our guidelines integrate evidence, expert consensus, and lived experience in 58 comprehensive recommendations for clinicians.
- These aim to improve diagnostic accuracy, early detection, and quality of care for people with cognitive disorders in Parkinson’s disease.

## Introduction

Parkinson’s disease (PD) is one of the fastest-growing neurodegenerative conditions, with disability and death due to PD increasing more rapidly than that reported in Alzheimer’s disease [1]. Although hallmark motor features define the disease, around 80% of people living with PD develop dementia by late stages of the disease [2]. In fact, cognitive impairment is a primary reason for institutionalisation in PD [3], and is linked to greater disability, lower quality of life, and higher carer stress [4]. Mild Cognitive Impairment in PD (PD-MCI) is a transitory state between pre-disease cognition levels and dementia. Approximately one quarter (21%–25%) of those with PD-MCI progress to PD dementia (PDD) within 5 years [5, 6], which is a more advanced state where the level of cognitive impairment is severe and functional independence is lost [7]. Early diagnosis of cognitive disorders is critical for providing timely and effective care [8]. However, differences in methods of neuropsychological assessment, access to neuropsychology resources, time taken to discuss cognitive impairment with patients, and adequate staff training across healthcare services produce delays in the diagnosis and management of cognitive disorders in PD [9–11]. Guidelines are thus needed to standardise best practice in this area. However, our systematic review revealed a distinct lack of up-to-date guidelines for cognitive disorders in PD [12], with the gold standard being published by the *Movement Disorder Society* (MDS) in 2012 and 2007 [7, 13]. More recently, *Parkinson’s UK* developed a Toolkit for Detecting and Managing Parkinson’s Dementia, offering practical guidance to clinicians for the identification and management of PDD [14]. However, there remains a need for multidisciplinary guidance that addresses early identification, structured screening, and comprehensive neuropsychological assessment across the full spectrum of cognitive disorders in PD. Here, we provide updated best practice guidelines for the diagnosis, evaluation and management of cognitive disorders in PD to standardise practice and promote evidence-based cognitive care for PD.

## Methodology

**Figure 1:**
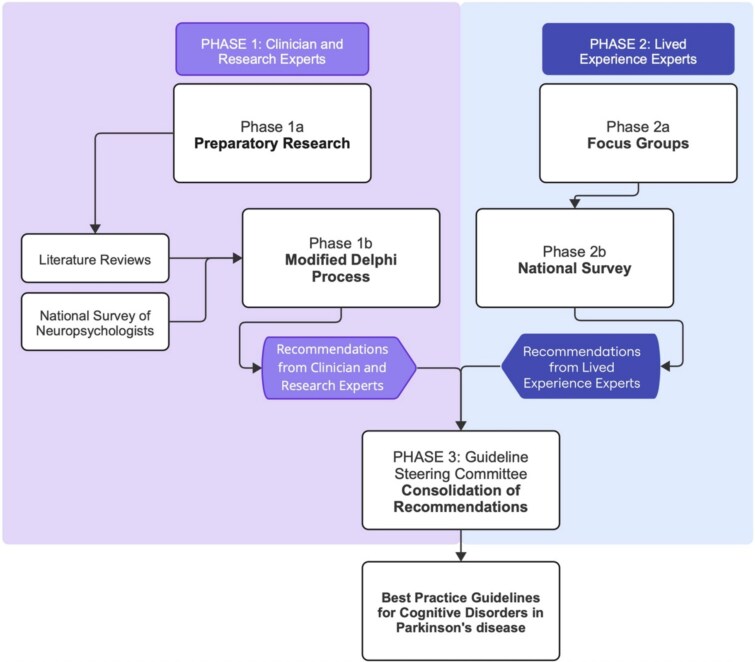
Methodology flow chart of the development of best practice guidelines.

Best practice guidelines were developed through consultation with clinicians and research experts, as well as people with lived experience of cognitive disorders in PD (lived experience experts). A flow chart of the methodology used to develop the recommendations within the best practice guidelines is provided in Figure 1. Further information on each phase is provided in the sections below.

### Phase 1: clinician and research expert consultation

Phase 1a (Figure 1: Phase 1a) of the clinician and research expert consultation involved preparatory research to inform initial evidence-based recommendations. Two literature reviews and a national survey were conducted. First, we completed a systematic review of guidelines and systematic reviews with recommendations for cognitive impairment in PD [12], synthesising current evidence of best practices for cognitive disorders in PD and highlighting critical gaps in the literature. Next, we conducted a systematic review to evaluate the evidence for cognitive tools predictive of cognitive decline in PD [15], ultimately informing the development of a standardised neuropsychological toolkit. Finally, a national survey of Australian neuropsychologists (*N* = 20) gauging current practices for identifying cognitive disorders in PD was conducted using a purposive sample from professional associations and networks. The survey collected information pertaining to clinical practices for people with PD, such as neuropsychological measures, diagnostic criteria, referral questions and teleneuropsychology. This information was used to ensure that the proposed recommendations align with current practice where empirical evidence was unavailable.

The preparatory research provided a summary of current evidence and critical gaps in the literature, informing the development of 58 initial recommendations for the diagnosis, evaluation, and post-diagnostic care of cognitive disorders in PD. These initial recommendations were then presented to a national panel of experts with clinical and/or research experience in cognitive disorders in PD to gather consensus in a two-round modified Delphi process (Phase 1b) [16]. In brief, 133 Australian clinician and research experts were contacted through purposive and snowball sampling to participate on the Delphi panel, with *N* = 29 experts participating in both rounds. Using a 5-point scale, agreement was defined as a median score ≥4 and consensus was defined as an Inter Quartile Rating (IQR) ≤1. Items with low agreement or consensus were revised after each round by a steering committee guided by written feedback from the panel. The Delphi procedure produced 51 final evidence-based and expert-informed recommendations for cognitive disorders in PD, inclusive of a comprehensive, PD-specific neuropsychological toolkit.

### Phase 2: lived experience expert consultation

Phase 2a (Figure 1: Phase 2a) of the lived experience expert consultation involved qualitative focus groups with people living with PD with subjective cognitive decline (*n* = 6), MCI (*n* = 3), dementia (n = 3), and their care partners (*N* = 3) [17]. Focus groups gathered participant perspectives on their lived experiences of cognitive evaluations, diagnosis of cognitive disorders, and post-diagnostic support for their cognitive symptoms. Qualitative analysis of these focus group discussions led to the development of 25 recommendations, which were then surveyed nationwide to gauge agreement (Figute 1: Phase 2b) amongst people living with PD and their care partners (*n* = 81). In total, 24 out of 25 recommendations achieved ≥70% agreement, median rating ≥4, and IQR ≤1 amongst the total sample and were included in the following best practice guidelines [17].

### Drafting of the best practice guidelines

Recommendations from both phases were consolidated by the PDCogniCare guideline steering committee, comprising a geriatrician (B.W.), movement disorders neurologist (J.O.S.), clinical psychologist and clinical neuropsychologist (L.K.M.), clinical psychologist and geropsychologist (N.A.P.), psychiatrist specialised in older persons (R.M.), speech pathologist (K.S.), occupational therapist (J.L.), pharmacist (E.T.), postdoctoral research fellow (D.P.), professorial research fellow (N.N.D.), person living with PD (W.K.M.), and care partner (N.P.). Upon completion of Phases 1 and 2 (Figure 1), the guideline steering committee convened in October 2024 to consolidate recommendations from both phases, with only minor changes suggested (Phase 3). The first draft of the best practice guidelines was prepared in January 2025 and revised by the guideline steering committee members in April–May 2025 to produce the final best practice guidelines (see: supplementary material).

### Strength of recommendations

Strong recommendation (SR): These recommendations are fundamental to the care of people living with cognitive disorders in PD. They were independently identified in the clinician and research expert and lived experience expert consultations, receiving endorsement from both groups.

Clinician and research expert recommendation (CR): These recommendations were endorsed by clinicians and research experts through the PDCogniCare Delphi procedure. It is expected that these recommendations can be met by all PD clinicians.

Lived experience expert recommendation (LR): These recommendations were developed and endorsed by lived experience experts. It is expected that these recommendations can be met by all PD clinicians.

Practice point (PP): Practice points represent ideal practices or important considerations for clinical care but may not be practical in all settings. These recommendations were derived from the clinician and research expert or lived experience expert consultations, but were identified by the Delphi panel or guideline steering committee to be potentially impractical in some settings.

### Intended users

These best practice guidelines have been developed for use in neurology and movement disorders clinics, where most Australians living with PD receive care. The intended users are professionals who work in movement disorders clinics, including neurologists, nurse practitioners, geriatricians, psychiatrists, clinical neuropsychologists, speech pathologists, and occupational therapists. The guidelines also serve as a comprehensive resource with up-to-date, evidence-based best practice recommendations to educate the next generation of medical and allied health clinicians.

The guidelines are intended to assist clinical decision-making because cognitive care may become a clinical priority ahead of motor function in later stages of the disease. However, they are not intended to be prescriptive or mandatory. Recommendations should be adapted to the clinical context, considering individual needs of each person living with PD. In doing so, it is intended that the guidelines will be applied with careful clinical judgement in collaboration with people living with PD and, where applicable, their care partners to optimise care.

## Recommendations

**Figure 2:**
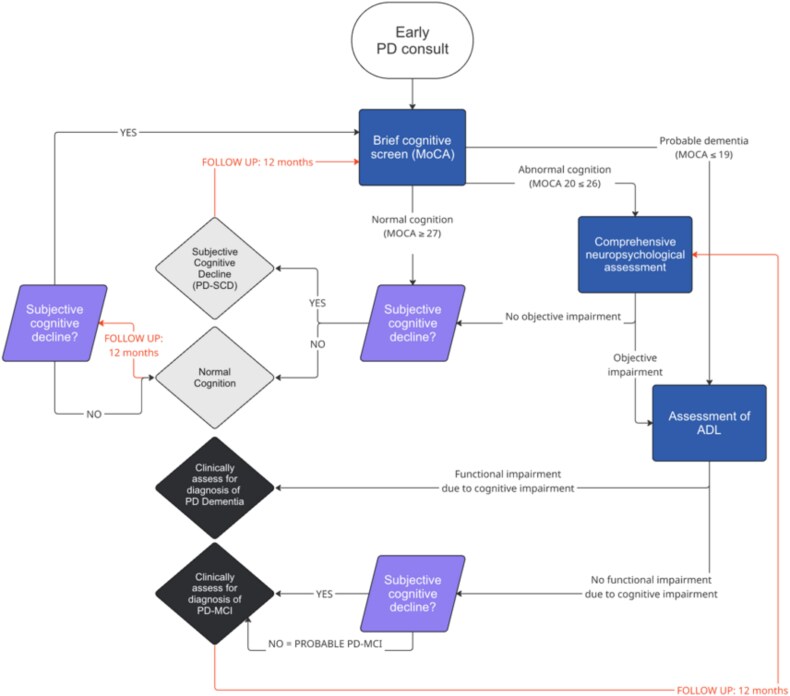
Decision-making flow chart for cognitive evaluations in PD. *Note.* ADL = Activities of Daily Living, MoCA = Montreal Cognitive Assessment, MCI = Mild Cognitive Impairment.

The best practice guidelines are provided in the supplementary materials. Recommendations within the guidelines are contained within four overarching sections. The first section, ‘*Who should receive a cognitive evaluation’,* pertains to the logistics of identifying people living with PD who should receive a cognitive evaluation in clinical settings. These recommendations aid clinicians in making important decisions in settings where neuropsychology resources are limited. Figure 2 provides a schematic flow chart of the recommendations provided in this section. *‘Diagnosis’* recommendations pertain to the diagnosis of cognitive disorders (mild neurocognitive disorder/MCI and major neurocognitive disorder/dementia) in PD. *‘Evaluation’* recommendations pertain to the selection and administration of tests for the cognitive evaluation of people living with PD. *‘Post-diagnostic care*’ recommendations pertain to best practices in post-diagnostic care for people living with cognitive disorders in PD, including treatment (pharmacological and non-pharmacological), support, and care planning.

Recommendations for an evidence-based neuropsychological toolkit were also produced to inform neuropsychological test choice for clinicians assessing cognitive impairment in PD. Measures included in the toolkit were chosen specifically for their utility in predicting cognitive decline in PD [15]. Currently, gold standard criteria for the diagnosis of cognitive disorders in PD include the MDS criteria for PD-MCI or PDD [7, 18], or Diagnostic Statistical Manual of Mental Disorders Fifth Edition- Text Revision (DSM-5-TR) criteria for minor or major neurocognitive disorder due to PD [19]. While the present best practice guidelines recommend application of the DSM-5-TR criteria for neurocognitive disorder due to PD, the toolkit aligns with both MDS and DSM criteria. Optimisation of the toolkit is ongoing, with the aim of validating the diagnostic accuracy of the toolkit, operationalising standardised parameters for identifying objective impairment, and exploring cost-effective applications of the toolkit in both face-to-face and telehealth modalities.

## Insights from recommendation development

Insights from the Delphi process and lived experience expert inquiry provide context for the best practice guidelines and highlight the rationale behind certain recommendations. The following section provides a summary of important findings from the development of the best practice guidelines that helped to shape the recommendations.

### Cognitive screening vs. comprehensive assessment

The Delphi process revealed a general sentiment towards conducting a baseline brief global cognitive screen soon after PD diagnosis [16]. The Montreal Cognitive Assessment was recommended as a brief screen due to its sensitivity to mild impairments in PD [20]. For those with no subjective cognitive decline, consider conducting a baseline screen as a measure of pre-morbid cognitive functioning. If a global cognitive screen indicates the presence of cognitive impairment, consider whether a comprehensive neuropsychological assessment is necessary to make a formal diagnosis. Comprehensive assessments may be lengthy, expensive, or distressing for some individuals, and these factors should be weighed against their clinical value in informing diagnosis, care planning, and patient and family support. For some people with severe impairment, the benefits may not justify the personal and financial expense. In these cases, consider conducting a needs assessment or functional assessment to guide care.

### Making the referral

The consumer and community inquiry revealed several insights regarding the referral pathways for cognitive evaluation in PD [17]. There was a general sentiment that allied health professionals may have regular contact with people living with PD and thus greater opportunities to detect cognitive decline over time. Allied health services are therefore encouraged to play a role in referrals for comprehensive neuropsychological assessment where possible. People living with PD who are referred for a comprehensive neuropsychological assessment should also be provided with sufficient information to make an informed decision. Potentially negative outcomes of comprehensive neuropsychological assessments may include a diagnosis of MCI or dementia, loss of legal or financial capacity, and loss of medical fitness to drive. Potentially positive outcomes of comprehensive neuropsychological assessments may include enhanced care, treatment of cognitive symptoms, future planning, and improved interpersonal relationships.

### Making the diagnosis

It is important to standardise methods for identifying cognitive disorders in PD to facilitate greater certainty in diagnostic decision-making [21]. While defining objective decline as a decrease from previous test scores is most accurate, it is not always feasible. Consider using estimated premorbid levels where previous testing is not available. Tele-neuropsychology should be offered, where feasible, to people who otherwise are unable to attend face-to-face neuropsychology assessments, given recent evidence for its reliability and validity in PD [22]. Cognitive subtyping should ideally be used to inform tailored treatments and therapies and identify participants eligible for clinical trials [23], but may be difficult to implement in a busy clinical setting. Finally, psychiatric evaluation is critical to the diagnosis of cognitive disorders in PD due to the symptom overlap between cognitive impairment and neuropsychiatric conditions such as depression and anxiety [24].

### Giving the diagnosis

Insights into the procedure of delivering a cognitive diagnosis were produced from both the Delphi process and lived experience expert inquiry [16, 17]. When diagnosis of a cognitive disorder occurs, written information should be provided to people living with PD and their care partners, as people experiencing cognitive impairment may struggle to process and recall information. The autonomy of people with cognitive disorders must also not be neglected. Similarly, it is important to collect appropriate consent prior to sharing sensitive information with others. The person living with PD should be given the opportunity to decide whether they wish to be present during discussions regarding diagnosis and neuropsychology test results, upholding the principles of autonomy, privacy, and dignity. Their decision should also be respected if they choose not to be present.

### Post-diagnostic care

The Delphi process and lived experience expert inquiry both revealed a strong sentiment for improved post-diagnostic care for people living with cognitive disorders in PD [16, 17]. In terms of pharmacological therapeutics, cholinesterase inhibitors were first-line for dementia with strong evidence to support their use [25]. However, clinicians recommended that transdermal application of rivastigmine should be considered in preference to oral administration to reduce gastrointestinal side effects and improve tolerability, although cost may also be a necessary consideration. Reducing polypharmacy in people with cognitive disorders was also recommended to improve adherence to medication and reduce the risk of cognitive side effects from various medications [26]. Physical exercise and cognitive rehabilitation demonstrated strong evidence to support their use for cognitive disorders in PD [27, 28], amongst other non-pharmacological interventions and lifestyle modifications. Finally, discussions around substitute decision making and advance care planning were recommended to be initiated as soon as possible, where feasible, after a cognitive diagnosis. In a sensitive yet timely manner, endeavour to discuss these topics with the person living with PD and their care partner before capacity is lost, without causing major distress to the patient.

## Implementation

Implementing the best practice guidelines in movement disorders clinics or other clinical settings where PD patients are seen will involve a structured approach to ensure accurate diagnosis and effective management of cognitive disorders. All clinical staff involved in PD care within a given setting should be informed of the recommendations and how to access them. Ideally, staff will receive training on key elements of the best practice recommendations related to their practice. For example, movement disorders nurses involved in conducting brief cognitive screens and referring patients for comprehensive neuropsychological assessment will receive specific advice on these procedures in accordance with the best practice guidelines. Training of multidisciplinary staff members involved in PD care will facilitate the establishment of clear referral pathways and care coordination both within and across clinical settings.

Information from pre-implementation interest holder interviews provided insight into barriers and facilitators for the implementation of best practice guidelines for cognitive disorders in PD [29]. An important barrier included limitations in clinic resources (e.g. staff, time, funding), which can hinder the ability to refer for or conduct comprehensive neuropsychological assessments and follow-ups. This is particularly relevant for busy or overburdened movement disorders clinics. Limitations in neuropsychology services were also identified as another potential barrier, particularly in regional and rural settings. In these cases, it may be challenging to implement some of the recommendations and provide training to staff on the appropriate use of the best practice guidelines. Another potential barrier to implementing the recommendations was the attitude towards cognitive evaluation, such as the perceived benefits of evaluations and how they inform patient management. Similarly, licencing, costing, and neuropsychologists’ personal preference for cognitive measures may be another barrier to adopting the recommended standardised toolkit within the best practice guidelines.

Conversely, facilitators to implementation of the recommendations include the development of a training package and mobilisation of clinical champions. Opportunity exists for integration of technology to enhance implementation of the guidelines by streamlining clinical referral processes and monitoring of cognitive decline over time [30]. The collaborative involvement of various interest holders in the development of the best practice guidelines to best meet their needs and expectations also increases the likelihood of their acceptance. This collaborative effort may also help promote awareness of best practice guidelines amongst PD clinicians and people with lived experience of PD.

From a health economics standpoint, systematic evaluation of these best practice guidelines is essential to demonstrate their clinical and economic value. Implementation should be assessed not only in terms of improved diagnostic accuracy, but also in terms of costs and benefits including improvements in quality of life, reductions in hospital admissions, delayed institutionalisation, reduced caregiver burden and more efficient use of health resources. Health economic modelling can provide robust evidence on the value of adopting these guidelines at scale. Embedding evaluation frameworks within real-world service delivery will allow payers and providers to quantify potential savings through earlier detection of cognitive decline, improved care coordination and better resource allocation.

## Limitations

Due to the evidence-led guideline development process, some clinical considerations fell outside the scope of the present guidelines. First, reversible iatrogenic factors that contribute to cognitive disorders in PD (e.g. anticholinergic burden) were not addressed. Second, risk factors of cognitive disorders in PD were not addressed, despite a recent study recommending tailored symptomatic treatments for high-risk groups [31]. These limitations reflect the scope of the guidelines and the evidence presented for Delphi consideration, rather than the clinical relevance of these important factors. Furthermore, it should be acknowledged that the guidelines function as a high-level, evidence-informed framework rather than a procedural manual. As such, recommendations are intended to be applied alongside existing disease- or intervention-specific guidelines and protocols. Finally, while the number of recommendations may pose challenges for busy clinicians, the guidelines are intended to serve as a comprehensive resource that multidisciplinary teams and services can selectively implement according to local needs, roles, and care contexts.

## Future directions

Recent advancements in biomarker discovery, Artificial Intelligence (AI)-driven diagnostics, and disease-modifying therapeutics for neurodegenerative disease provide insight into the potential future of cognitive research in PD. Although the guideline development process did not reveal sufficient evidence to support clinical recommendations in these fields, future iterations should focus closely on these rapidly evolving areas. Biomarkers aim to enhance early diagnosis, prognosis, and treatment of cognitive disorders in PD. Emerging literature exploring cerebrospinal fluid, imaging, and blood biomarkers shows promise for early identification of PDD [32]. AI-driven models have also demonstrated potential to integrate multimodal data to improve diagnostic accuracy, predict cognitive decline, identify subtypes of cognitive impairment, and personalise and optimise treatment interventions [33, 34]. Finally, scientific breakthroughs in disease-modifying therapeutics such as monoclonal antibodies for the treatment of Alzheimer’s disease and gene therapy targeting PD motor symptoms instil hope for future treatment of cognitive disorders in PD [35, 36]. As these technologies evolve, so too will the best practice guidelines. Regular updates to the recommendations will be necessary to maintain current, evidence-based practices and a high quality of care for people living with PD.

Beyond advances in research, implementation and evaluation of the best practice guidelines must be conducted to determine their cost-effectiveness and scalability in clinical settings. International collaboration will be critical to promote the standardisation of clinical practice and quality of care across PD populations globally. While some recommendations such as referral pathways, neuropsychology practices, and referral to psychology for distressed patients may be unique to the Australian healthcare setting, many recommendations are globally relevant and readily transferable across healthcare contexts, such as screening protocols, diagnostic frameworks, and care considerations. To ensure sustainable implementation of the guidelines, integration into existing clinical pathways, training of multidisciplinary teams, and alignment with funding structures will be necessary. Future service models should prioritise scalable approaches, including the use of digital health and tele-neuropsychological assessments, to improve accessibility in regional and resource-constrained settings. Partnerships with policymakers, insurers, and patient advocacy organisations will be critical in embedding these guidelines into standard care. Health economic analyses can further guide prioritisation of resource allocation, ensuring that the guidelines deliver value for money within ageing health systems facing rising demand.

## Conclusion

Our evidence-based and expert-endorsed best practice recommendations for the diagnosis, evaluation, and management of cognitive disorders in PD work towards improving the quality and consistency of care across clinical settings. With input from all relevant interest holders, these best practice guidelines will facilitate the standardisation of cognitive evaluations and neuropsychology methods to improve awareness and identification of cognitive disorders, with a view to improving both quality of care and quality of life for people living with PD.

## Supplementary Material

aa-25-3298-File002_afag063

## Research Data Transparency and Availability:

Data may be shared upon request.
