# Supplementary material for: Best practice guidelines for the diagnosis, evaluation, and management of cognitive disorders in Parkinson’s disease
Source: Age Ageing. 2026 Mar 23;55(3):afag063. doi: 10.1093/ageing/afag063 (PMC13016819; doi:10.1093/ageing/afag063)
Supplement: aa-25-3298-File002_afag063 [file aa-25-3298-file002_afag063.pdf]

BEST PRACTICE GUIDELINES

for the Diagnosis, Evaluation and Management of Cognitive Disorders in Parkinson's disease

PDCogniCare

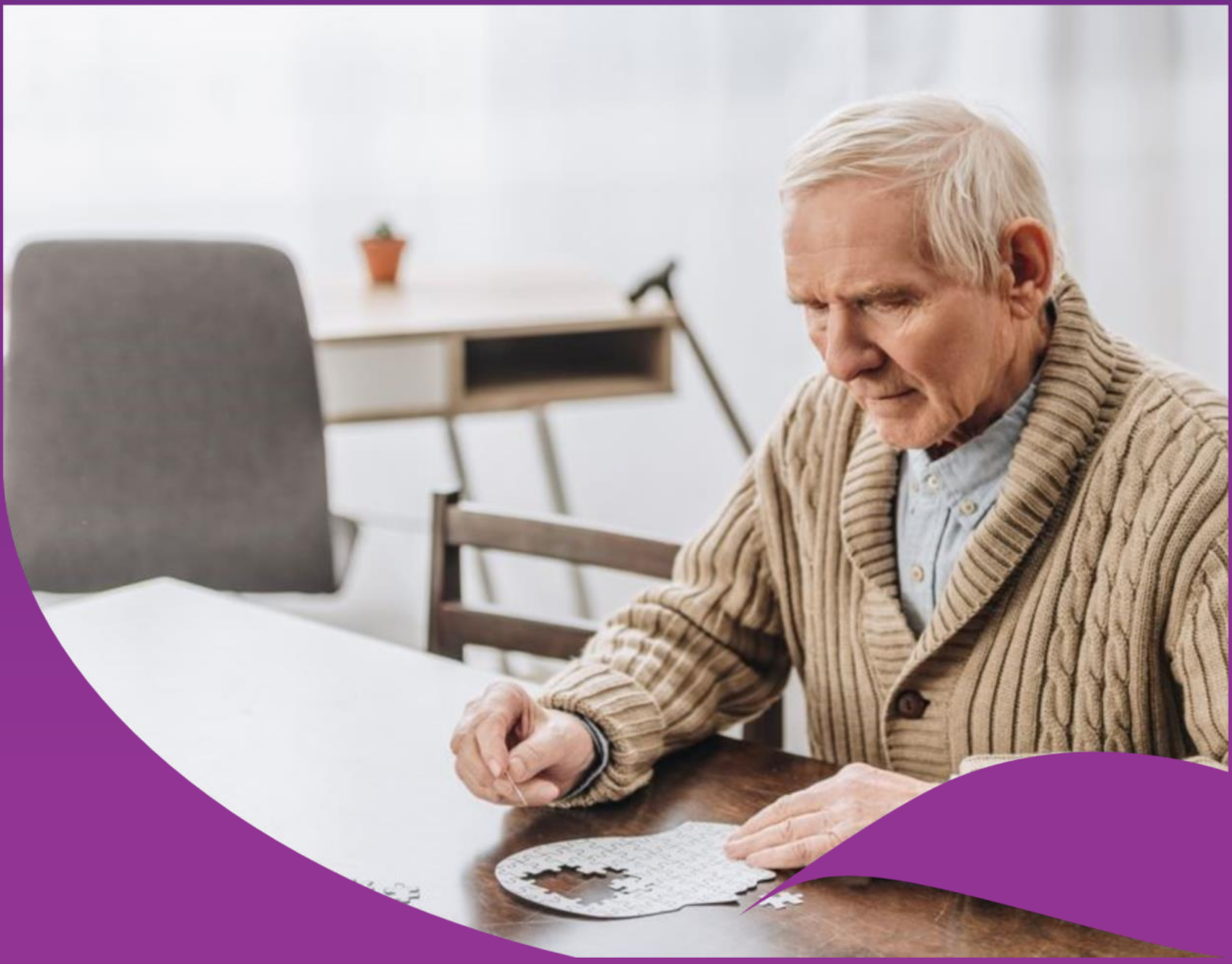

PDCogniCare
Best Practice Guidelines (V2.0 dated February 9, 2026)

Principal Investigator

Professor Nadeeka Dissanayaka
The University of Queensland

**Address**	The University of Queensland Centre for Clinical Research (UQCCR)<br>Building 71/918,<br>Royal Brisbane & Women's Hospital Campus.<br>Herston QLD 4029, Australia
**Telephone**	+61 7 3346 6026<br>+61 7 33465577
**Email**	pdcognicare@uq.edu.au
**Website**	https://clinical-research.centre.uq.edu.au/pdcognicare
**Funding**	MRFF Dementia Ageing and Aged Care Mission Grant 2022

Contents

1 Introduction 9

1.1 Background 9

1.2 Rationale 10

1.3 Objectives 10

1.4 Clinical population 11

1.5 Intended users 11

1.6 Intended use 11

2 Methods 12

2.1 Phase A: Clinician & Research Expert Consultation 12

2.2 Phase B: Lived Experience Expert consultation 13

2.3 Drafting of the guidelines 13

2.4 Update of the guidelines 13

2.5 Strength of recommendations 14

3 Recommendations 15

3.1 Who should receive a cognitive evaluation 15

3.2 Diagnosis 18

3.3 Evaluation 21

3.4 Post-diagnostic care 25

4 Neuropsychological Toolkit 29

4.1 Recommended measures 29

4.2 Practical considerations 30

4.3 Intended use 29

5 Dissemination 31

5.1 Implementation 31

5.2 Barriers and Facilitators 31

6 Future directions 32

7 References 33

Glossary

**Allied Health**	Health professionals such as dietitians, nutritionists, exercise physiologists, occupational therapists, physiotherapists, psychologists, social workers and speech pathologists. These clinicians prevent, diagnose, and treat Parkinson's disease symptoms, and support function, independence and quality of life for people living with Parkinson's disease.
**Brief cognitive screen**	Tests that provide a global measure of cognition. They evaluate multiple cognitive domains in a single assessment and take 5-10 minutes to complete. Any appropriately trained clinician or researcher can administer a brief cognitive screen.
**Capacity**	A legal concept defining an individual's ability to understand and evaluate information to make informed decisions about their own medical care. It evaluates a person's cognitive and emotional ability to process relevant information, weigh potential risks and benefits, communicate choices, and understand the consequences of those choices.
**Care partner**	Someone who provides care to a person living with Parkinson's disease. This person may be paid or unpaid, and typically provides assistance with activities of daily living.
**Cautious deprescribing**	The systematic process of discontinuing or reducing the dose of medications to prioritise patient safety where the risks of polypharmacy may outweigh the benefits.
**Cognitive evaluation**	A broad term for the assessment of cognitive symptoms. Evaluations can involve clinical interviews, standardised tests, and/or general observations. Two types of evaluations referred to in this document include: brief cognitive screens and comprehensive neuropsychological assessments.
**Comprehensive neuropsychological assessment**	Cognitive evaluations conducted by a clinical neuropsychologist. Various domains of cognition are assessed. Related psychological symptoms may also be assessed. These assessments may take several hours to complete and can be used to diagnose cognitive disorders and inform intervention and care plans.

**Dementia in Parkinson's disease**	A cognitive disorder defined by objective impairment on cognitive tests concurrent with impairment in activities of daily living. Dementia in Parkinson's disease tends to have a different presentation and aetiology compared to other dementias.
**Feedback session**	An appointment conducted by neuropsychologists to provide feedback to their clients on their cognitive test performance and/or cognitive status.
**Informant**	A family member, care partner, or close acquaintance of a person living with Parkinson's disease who provides information about their cognitive, functional, and behavioural changes over time.
**Lived Experience Expert**	Someone with lived experience of Parkinson's disease. This includes people living with Parkinson's disease and their care partners.
**Mild cognitive impairment in Parkinson's disease**	A cognitive disorder defined by mild impairments in cognitive function. It is a transitory phase between pre-disease cognition and dementia, where cognition is impaired but activities of daily living have not yet been impacted.
**Post-diagnostic care**	Support and advice provided to people living with Parkinson's disease after a cognitive diagnosis is made, including access to psychoeducation, interventions and treatments, and information and services to improve quality of life.
**Telehealth cognitive evaluations**	Cognitive evaluations that are delivered remotely, either over the phone or via video-conferencing platforms.

Abbreviations

**ADL**	Activities of Daily Living
**CCIG**	Consumer and Community Involvement Group
**DBS**	Deep Brain Stimulation
**DLB**	Dementia with Lewy Bodies
**DSM-5-TR**	Diagnostic Statistical Manual of Mental Disorders - Fifth Edition - Text Revision
**FDG-PET**	Fluorodeoxyglucose Positron Emission Tomography
**GP**	General Practitioner
**LBD**	Lewy Body Disease
**MDS**	Movement Disorder Society
**MoCA**	Montreal Cognitive Assessment
**OT**	Occupational Therapy
**PD**	Parkinson's disease
**PD-MCI**	Mild Cognitive Impairment in Parkinson's disease
**PDD**	Dementia in Parkinson's disease

Steering Panel Members

Panel Member	Role
**Prof Nadeeka Dissanayaka**	PDCogniCare Principal Investigator
**Dr Deborah Brooks**	PDCogniCare Project Coordinator<br>Research Fellow
**Dr Dana Pourzinal**	Guideline Development Lead<br>Post-doctoral Research Fellow
**Dr Deepa Sriram**	Consumer and Community lead<br>Post-doctoral Research Fellow
**Dr Emily McCann**	Toolkit Development Lead<br>Post-doctoral Research Fellow
**Mr James King**	Telehealth Development Lead<br>PhD Candidate
**Em Prof Neil Page**	Lived Experience Expert
**Em Prof Kim Halford**	Lived Experience Expert
**Prof Brian Wood**	Consultant Geriatrician and General Physician
**A/Prof John O'Sullivan**	Consultant Neurologist specialised in Movement Disorders
**Dr Rodney Marsh**	Consultant Psychiatrist specialised in Old Age Psychiatry
**Prof Nancy A. Pachana**	Clinical Geropsychologist
**Dr Leander K. Mitchell**	Clinical Neuropsychologist and Clinical Psychologist
**Dr Kirstine Shrubsole**	Speech Pathologist
**A/Prof Jacki Liddle**	Occupational Therapist
**Dr Edwin Tan**	Pharmacist

Acknowledgements

We acknowledge the contributions of the overarching PDCogniCare team: Tiffany Au, Prof Elizabeth Beattie, A/Prof Annette Broome, Prof Gerard Byrne, Mark Chatfield, Anna Kelder, Dr Syed Afroz Keramat, A/Prof Alexander Lehn, Dr Elton Lobo, Prof Sharon Naismith, Prof Peter Nestor, Stuart Robertson, Dr Kumar Sivakumaran, Dr Donna Spooner, Prof Martie-Louise Verreynne, Dr Peter Worthy, and Dr Jihyun Yang. We also extend our gratitude to the many stakeholders who have made significant contributions to these guidelines. This includes the Australian Dementia Network (ADNeT), Dementia Australia, Parkinson's Queensland Incorporated, Metro North and Metro South Health Services in Brisbane, Australia, and our community partners from the Lions Q3 District, Lions Club of Brisbane Inner North, and the Consumer and Community Involvement Group (CCIG). This also includes the clinician and research experts across Australia who contributed to the Delphi panel to inform the development of the guidelines. We would also like to thank the many people living with Parkinson's disease and their families, care partners, and clinicians across Australia for their meaningful contributions to the focus groups, surveys, and consultations required of the project and for their efforts to help shape these guidelines.

Funding

The PDCogniCare project was funded by the Australian Government National Health and Medical Research Council (NHMRC) Medical Research Futures Fund (MRFF) Dementia and Aged Care (DAAC) grant scheme (MRF2023746). This grant funded research and development of the guidelines for the diagnosis, evaluation, and management of cognitive disorders in Parkinson's disease. However, the granting body did not influence the decisions reached or play a role in the development of these guidelines.

Disclaimer

Authors: Professor Nadeeka Dissanayaka and Dr Dana Pourzinal.
The University of Queensland, 2025.

These guidelines were developed by the PDCogniCare team. The recommendations for diagnosis, evaluation, and management of cognitive disorders in Parkinson's disease and accompanying guidelines have been developed as part of the "Enhancing Utility of Neuropsychological Evaluation for Earlier and Effective Diagnosis of Dementia in Parkinson's disease" PDCogniCare project funded by Medical Research Future Fund. These guidelines have not been developed to tailor for Australian First Nations Peoples or culturally and linguistically diverse communities.

1 Introduction

1.1 Background

Dementia is the leading cause of disease burden in Australian older adults and can necessitate high levels of care, particularly in terms of hospitalisation and institutionalisation at late stages.[1] For this reason, the economic cost of dementia is cumbersome at >$14 billion per annum, which is projected to double by 2050 due to the ageing population.[2] Reducing the impact of dementia on the economy, to society, and to the individual is contingent on accurate identification of cognitive impairment at its earliest stages to guarantee timely provision of treatment and care. However, for people living with Parkinson's disease (PD), the cognitive symptoms of dementia are often overlooked in clinical practice.

PD is one of the fastest-growing progressive, neurodegenerative conditions, with disability and death due to PD increasing more rapidly than that of Alzheimer's disease.[3] Although hallmark motor features define the disease, cognitive impairment and dementia are highly prevalent throughout the course of PD, with 80% of people living with PD developing dementia by late stages of the disease.[4] In fact, cognitive impairment is a primary reason for institutionalisation in PD,[5] and is linked to greater disability, lower quality of life, and higher carer stress in people living with PD.[6] There is also a direct link between cognitive decline and increased healthcare expenses for the person living with PD, with greater cognitive impairment leading to higher incurred costs.[7] While there are no estimated costs reported for PD dementia, in Australia, the annual total cost of PD is $9.8 billion.[8]

The diffuse and heterogeneous spread of alpha-synuclein aggregates called Lewy bodies in the cerebral cortex and limbic structures are the main pathological substrates of dementia in PD.[9] This may in part explain the vast heterogeneity in the presentation and progression of cognitive symptoms in PD. People living with PD can experience various cognitive symptoms, including deficits in memory, language, visuospatial function, executive function, and attention/working memory.[9] Magnetic Resonance Imaging (MRI) studies have demonstrated that these deficits are underpinned by heterogenous changes in neural substrates.[10-12] The rate at which people living with PD progress toward cognitive decline also varies significantly, with some demonstrating gradual decline over many years and others deteriorating rapidly.[4, 13] Despite this heterogeneity, all cognitive impairments impact quality of life for people living with PD.[14]

There are two identifiable cognitive disorders in PD. Mild Cognitive Impairment in PD (PD-MCI) is a transitory state between pre-disease cognition levels and dementia. PD-MCI is prevalent in approximately one quarter (25-27%) of people living with PD.[15, 16] During this phase, which can be transient and unstable,[17] impairments are mild and

functional ability remains intact.[18] Clinical subtypes may also be evident during this phase with distinct presentations of cognitive symptoms and rate of cognitive decline.[19] Approximately one quarter (21-25%) of those with PD-MCI progress to PD dementia (PDD) within five years,[20, 21] which is a more advanced state where cognitive impairments are severe and functional independence is lost.[22] PD-MCI and PDD are analogous to the ‘mild and major neurocognitive disorder due to PD’ classifications in the Diagnostic Statistic Manual of Mental Disorders Fifth Edition- Text Revision (DSM-5-TR),[23] respectively.

1.2 Rationale

Early diagnosis of cognitive impairment is critical for providing timely and effective care.[9] However, the diagnosis, evaluation and management of cognitive disorders in PD can vary significantly from clinic to clinic. Systematic differences in clinical practice above and beyond patient-centred care can impact the quality of care. For example, variability in the methodology used to identify cognitive disorders, such as choice of neuropsychological measures and diagnostic criteria, can impact on who receives a diagnosis.[24, 25] Furthermore, cognitive evaluation is not routinely available or integrated into primary health care, specialist neurology or geriatric clinical services.[26] While some clinics may be able to facilitate in-house comprehensive neuropsychological assessments, others fail to even broach the topic of cognitive impairment with patients due to time constraints, limited resources, and/or inadequate staff training.[27]

Poor management of cognitive deficits in PD therefore creates delays in the diagnosis of cognitive disorders, leading to subsequent delays in treatment, rehabilitation potential, support, and future planning. Current practice does not meet the needs of people living with cognitive disorders in PD, who report the feeling of "falling through the gaps" in the healthcare system.[28] While excellent resources for the diagnosis of PD exist, guidelines for the evaluation, diagnosis and management of cognitive disorders in PD are lacking. The most recent gold standard guidelines for PD-MCI and PDD were published by the *Movement Disorder Society* in 2012 and 2007.[17, 22] Updated guidelines are needed to educate relevant staff on current best practices for cognitive disorders in PD, to harmonise and streamline cognitive evaluation across PD clinics, and to provide people living with PD with optimal care.

1.3 Objectives

The objectives of the current guidelines are to provide evidence-based recommendations backed by Australian experts for the diagnosis, evaluation and management of cognitive disorders in PD. This includes recommendations for a harmonised neuropsychological toolkit to standardise methods of identifying cognitive disorders in PD across clinics, as well as a telehealth toolkit to facilitate neuropsychological services for people in regional, rural, or remote settings and those with mobility issues.

1.4 Clinical population

These guidelines provide recommendations for cognitive disorders in people living with PD. They are not intended for other parkinsonian disorders, such as Dementia with Lewy Bodies (DLB) or other conditions. This boundary was set deliberately to ensure clarity for intended users and to align recommendations with the clinical context of PD care pathways. However, we acknowledge the substantial clinical overlap between PD and other conditions, and that some individuals initially diagnosed with PD may later be reclassified (e.g., with DLB) or vice versa. In such cases, these guidelines are relevant to the evaluation of cognitive symptoms occurring within PD care settings, but they are not intended to substitute for disease-specific (e.g., DLB-specific) diagnostic or management guidance.

1.5 Intended users

PDCogniCare guidelines have been developed for use in movement disorders clinics, where Australians living with PD primarily receive care. The intended users are movement disorders clinicians such as neurologists, nurse practitioners, and geriatricians, as well as other clinicians involved in PD care such as psychiatrists, clinical neuropsychologists, speech pathologists, and occupational therapists. In a broader sense, however, we intend for these guidelines to inform the diagnosis, evaluation and management of cognitive disorders in PD in various clinical settings where cognitive disorders are diagnosed, such as hospitals, private clinics, GP clinics, residential aged care, and community health services.

1.6 Intended use

These guidelines provide evidence-based recommendations to support clinical decision-making for diagnosing and managing cognitive disorders in PD. They are not intended to be prescriptive or mandatory. Recommendations should be adapted to the clinical context, taking into account individual circumstances, values, and preferences of each person living with PD. In doing so, it is intended that the recommendations will be applied with careful clinical judgment in collaboration with people living with PD and, where applicable, their care partners to optimise care.

2 Methods

These guidelines were developed through consultation with clinician and research experts, as well as people with lived experience of cognitive disorders in PD (lived experience experts). A flow chart of the methodology used to develop the guidelines is provided in Figure 1. Further information on each phase is provided in the sections below.

Figure 1. Flow-Chart of the guideline development process

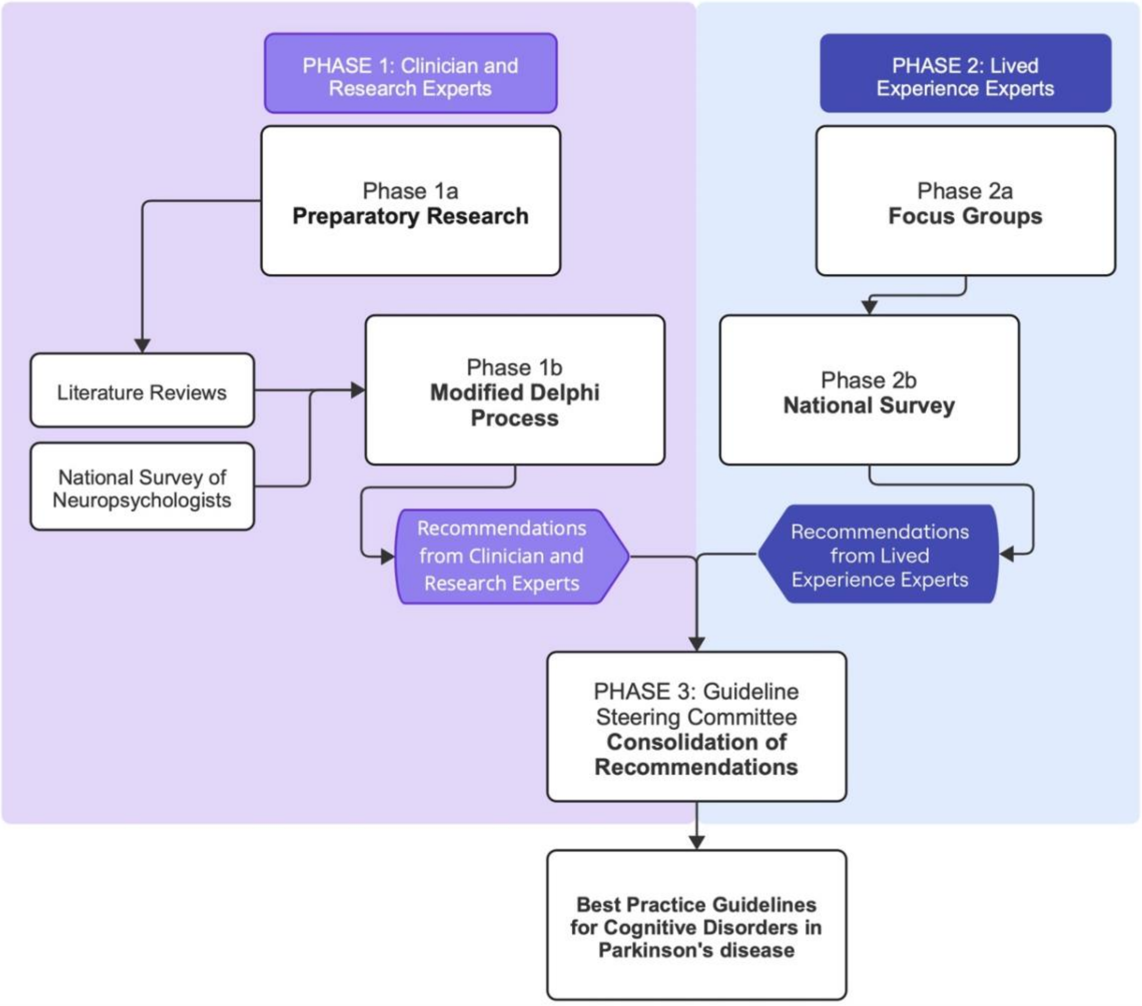

2.1 Phase 1: Clinician & Research Expert Consultation

Phase 1a (Figure 1) of the clinician and research expert consultation involved preparatory research to inform initial evidence-based recommendations. Two systematic literature reviews were conducted: a review of guidelines and systematic reviews with recommendations for cognitive impairment in PD,[30] and a review of cognitive tools used to predict cognitive decline in PD.[31] A national survey of Australian neuropsychologists (N=20) gauging current practices for cognitive impairment in PD was also conducted to ensure that the proposed recommendations align with current

practice where empirical evidence was unavailable. The preparatory research provided a summary of current evidence and critical gaps in the literature, informing the development of 58 initial recommendations for the diagnosis, evaluation, and post-diagnostic care of cognitive disorders in PD. These initial recommendations were then presented to a national panel (N=29) of experts with clinical and/or research experience in cognitive disorders in PD in a two-round modified Delphi process (Phase 1b).[32] The Delphi procedure produced 51 final evidence-based and expert-backed recommendations for cognitive disorders in PD.

2.2 Phase 2: Lived Experience Expert consultation

Phase 2a (Figure 1) of the lived experience expert consultation involved qualitative small group discussions with N=15 people living with PD with subjective cognitive decline, mild cognitive impairment, dementia, and their care partners. Focus groups gathered participant perspectives on their lived experiences of cognitive evaluations, diagnosis of cognitive disorders, and post-diagnostic support for their cognitive symptoms. Qualitative analysis of the focus group discussions led to the development of 25 recommendations, which were then surveyed nationwide (Phase 2b) among people with PD and their care partners (N=81). Twenty-four recommendations achieved ≥70% agreement among the total sample and were included in the guidelines.[33]

2.3 Drafting of the guidelines

Upon completion of Phase 1 and Phase 2, the PDCogniCare Delphi Steering Committee convened in October 2024 to consolidate recommendations from both expert groups, with only minor changes suggested (Phase 3). The first draft of the best practice guidelines was prepared in January 2025 and revised by the PDCogniCare Steering Committee members in April-May 2025. A final version of the guidelines was derived.

2.4 Update of the guidelines

Guidelines will be updated every five years to maintain currency. Recommendations should be revised based on emerging evidence, align with the latest clinical practices and policy changes, and incorporate new technologies and treatment approaches.

2.5 Strength of recommendations

The recommendations provided in these guidelines adhere to the following system:

Recommendation		Definition
**SR**	Strong recommendation	These recommendations are fundamental to the care of people living with cognitive disorders in PD. They were independently raised in both the clinician and research expert, and lived experience expert streams of inquiry, with endorsement from both groups.
**CR**	Clinician and research expert recommendation	These recommendations were endorsed by clinician and research experts through the PDCogniCare Delphi procedure. It is expected that these recommendations can be met by all PD clinicians.
**LR**	Lived experience expert recommendation	These recommendations were developed and endorsed by lived experience experts. It is expected that these recommendations can be met by all PD clinicians.
**PP**	Practice Point	Practice points represent ideal practices or important considerations for clinical care but may not be relevant or feasible for all clinics and cases.

3 Recommendations

3.1 Who should receive a cognitive evaluation

The following recommendations pertain to the logistics of identifying people living with PD who should receive a cognitive evaluation in clinical settings. Figure 2 at the end of this section provides a schematic flow chart of the information provided.

3.1 Why is this important?
The standardisation of cognitive evaluations in PD will improve awareness and the identification of cognitive disorders in this population. These recommendations provide guidance as to who should receive a cognitive evaluation to aid clinicians in making critical decisions in settings where resources are limited.

Initial screen

SR

3.1.1 People with PD and their care partners should be asked to report on subjective cognitive decline (i.e., perceived problems with memory or thinking) at the time of PD diagnosis.

SR

3.1.2 Consider a brief global cognitive screen (e.g., Montreal Cognitive Assessment) for all people living with PD shortly after diagnosis of PD as a baseline measure of cognition, regardless of subjective cognitive decline.

3.1.2 Why is this important?
Conducting a baseline brief global cognitive screen may be useful to track changes in cognition over time. For those with no subjective cognitive decline, consider conducting a baseline screen as a measure of pre-morbid cognitive functioning.

Follow up screen

SR

3.1.3 All people living with PD and their care partners should be asked to report on subjective cognitive decline (i.e., perceived problems with memory or thinking) at clinical review at least ***every 12 months****.*

CR

3.1.4 If subjective cognitive decline is reported ***at a follow up appointment****, administer a brief global cognitive screen (e.g., Montreal Cognitive Assessment).*

SR

*3.1.5 People living with PD who show **intact** global cognitive function on a brief screen (e.g., Montreal Cognitive Assessment ≥ 27) and report subjective cognitive decline should be considered for a repeat **brief** cognitive screen within 12 months.*

Initial comprehensive neuropsychological assessment

CR

*3.1.6 A comprehensive neuropsychological assessment should be considered for people living with PD who show **reduced** global cognitive function on a brief screen (e.g., Montreal Cognitive Assessment 20 ≤ 26).*

PP

3.1.7 People living with PD who show significant impairment in global cognitive function on a brief screen (e.g., Montreal Cognitive Assessment ≤ 19) may be less likely to tolerate comprehensive neuropsychological assessment. Carefully consider the purpose and overall benefit of assessment in these cases.

3.1.7 Practice point:
Comprehensive neuropsychological assessments may take several hours to complete, can be expensive, and may be distressing for some. For people with severe impairment, the benefits of comprehensive assessment may not justify the personal and financial expense. Carefully consider whether a comprehensive assessment is necessary to make a formal diagnosis.

CR

*3.1.8 If significant impairment in global cognitive function on a brief test (e.g., Montreal Cognitive Assessment ≤ 19) and functional impairment is present, clinically assess for a diagnosis of **dementia**.*

Follow up comprehensive neuropsychological assessment

SR

*3.1.9 People living with PD who are diagnosed with **mild cognitive impairment** after comprehensive neuropsychological assessment should be considered for a repeat comprehensive assessment within 12 months.*

SR

*3.1.10 People living with PD who are diagnosed with **mild dementia** after comprehensive neuropsychological assessment should be scheduled for a repeat comprehensive neuropsychological assessment at the discretion of the clinician, the person with PD, and their care partners.*

PP

3.1.11 People living with PD who are diagnosed with ***moderate to severe dementia*** *after comprehensive neuropsychological assessment are less likely to tolerate or benefit from repeat comprehensive neuropsychological assessment. Consider a needs assessment to guide care.*

3.1.11 Practice point:
Comprehensive neuropsychological assessments may take several hours to complete, can be expensive, and may be distressing for some. For people with severe impairment, the benefits of comprehensive assessment may not justify the personal and financial expense. In these cases, consider conducting a needs assessment or functional assessment to guide care.

Figure 2. Decision-making flow chart for cognitive evaluations in PD.

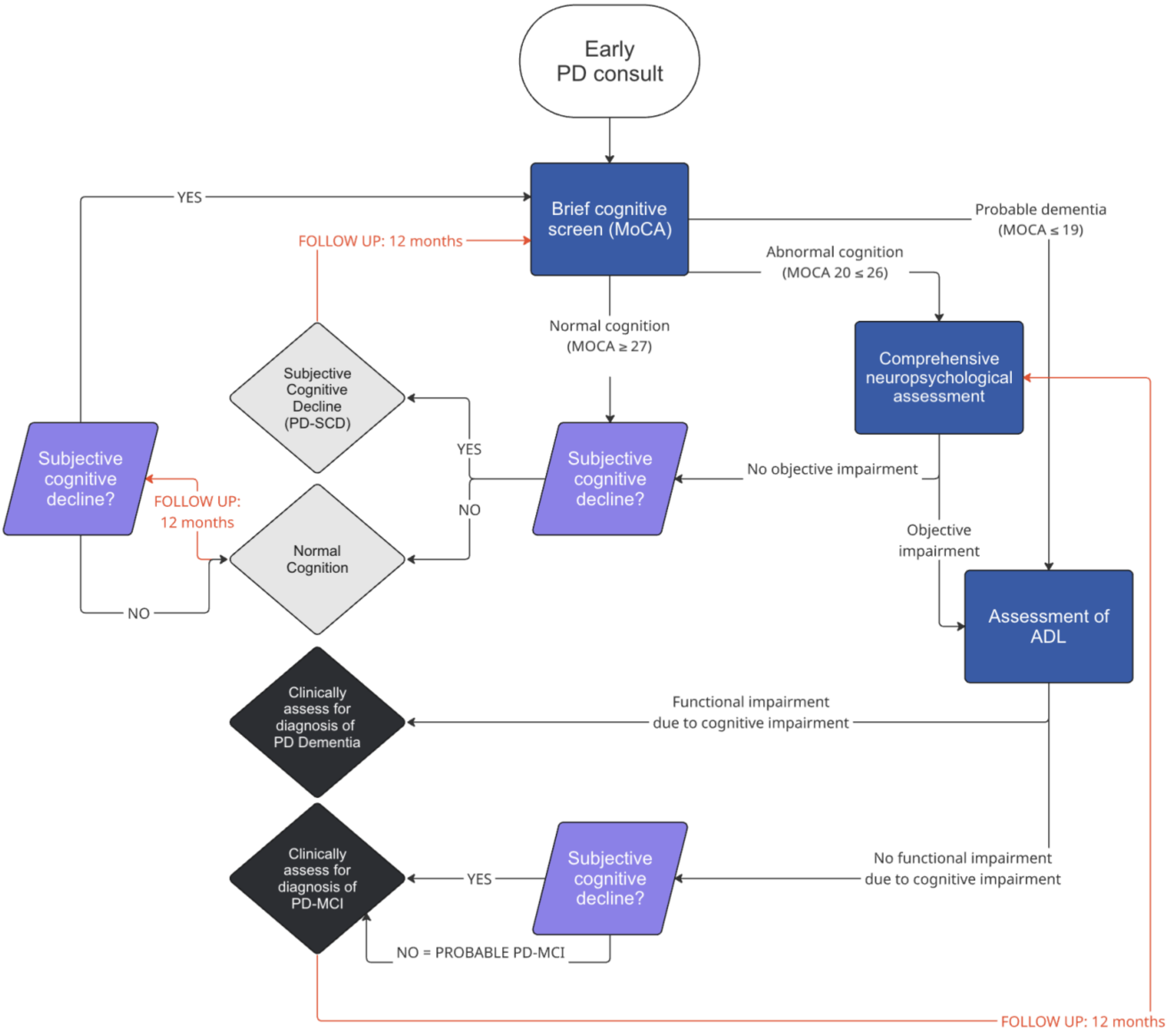

Note. ADL = Activities of Daily Living, MoCA = Montreal Cognitive Assessment, MCI = Mild Cognitive Impairment

3.2 Diagnosis

Diagnosis of cognitive disorders may be made by various clinicians involved in the care of people living with PD. The following recommendations pertain to the diagnosis of cognitive disorders (mild neurocognitive disorder / mild cognitive impairment and major neurocognitive disorder / dementia) in PD.

Diagnostic criteria

CR

3.2.1 DSM-5-TR criteria for major neurocognitive disorder (dementia) due to PD and mild neurocognitive disorder (mild cognitive impairment) due to PD should be used to diagnose cognitive disorders.

CR

3.2.2 Objective decline should be defined as >1SD decrease (based on relevant norms) in performance from previous testing, or >1SD decrease from estimated premorbid levels where previous testing is unavailable.

3.2.2 Why is this important?
It is important to standardise methods for identifying cognitive disorders in PD to allow for greater certainty in diagnostic decision making. While defining objective decline as a decrease from previous test scores is most accurate, it is not always feasible. Consider using estimated premorbid levels where previous testing is not available.

CR

3.2.3 The following tests may be used to explore alternative causes of cognitive impairment prior to a diagnosis of mild cognitive impairment in PD:

Psychiatric evaluation (depression, anxiety, hallucinations)
Blood tests (vitamin B12, folate, TSH, liver function, anti-neuronal antibody panel, syphilis, zoonoses, and HIV)
Magnetic Resonance Imaging (MRI) or computed tomography (CT) (vascular dementia, stroke and brain tumours)

CR

3.2.4 The following tests may be used to rule out alternative causes of cognitive impairment prior to a diagnosis of dementia in PD:

Psychiatric evaluation (depression, anxiety, hallucinations)
Blood tests (vitamin B12, folate, TSH, liver function, anti-neuronal antibody panel, syphilis, zoonoses, and HIV)
Magnetic Resonance Imaging (MRI) or computed tomography (CT) (vascular dementia, stroke and brain tumours)
Fluorodeoxyglucose (FDG)-Positron Emission Tomography (PET) (cortical hypometabolism)

3.2.4 Practice point:
FDG-PET is recommended where possible but is not always feasible in settings with limited resources. SPECT imaging may be an appropriate alternative.

PP

3.2.5 Where applicable, cognitive subtypes (e.g., amnestic, frontal dysfunction) should be identified to inform care procedures (e.g., tailored cognitive rehabilitation), psychoeducation, and entry into clinical trials.

3.2.5 Practice point:
Ideally, cognitive subtyping should be used to inform tailored treatments and therapies and identify eligible participants for clinical trials. However, cognitive subtyping may require additional analysis of neuropsychological test results and thus may be difficult to implement in a busy clinical setting

Delivery of diagnosis

SR

3.2.6 Diagnosis of cognitive disorders should be clearly communicated verbally to people living with PD and their care partners with written information provided for personal review.

3.2.13 Why is this important?
People with cognitive impairment may struggle to process and recall information. It is critical to provide written information to both people living with PD and their care partners for their perusal at home.

SR

3.2.7 Diagnosis of cognitive disorders should be delivered to people living with PD in a sensitive and empathetic manner.

LR

3.2.8 Clinicians should ask people living with PD if they would like an in-depth discussion about what a cognitive diagnosis means for different areas of their life (such as the short, medium and long-term outlook).

Consent and confidentiality

LR *3.2.9 Consider sharing neuropsychological test results with a primary care physician (e.g., GP) and allied health services that may benefit from this information (e.g., speech pathology, physiotherapy, occupational therapy).*

CR *3.2.10 People living with PD who receive a cognitive diagnosis should be asked if and with whom the outcome of their comprehensive neuropsychological assessment may be shared.*

3.2.18 Why is this important?
The autonomy of people with cognitive disorders must not be neglected. It is important to collect appropriate consent prior to sharing sensitive information.

3.3 Evaluation

There are unique considerations for cognitive evaluations for people living with PD. The following recommendations pertain to the administration and selection of tests for the cognitive evaluation of people living with PD.

Indication for cognitive evaluation

3.3.1 People living with PD should be considered for comprehensive neuropsychological assessment:

**(SR)** If subjective cognitive decline or decline in functional ability is perceived by the person living with PD or informant.
**(CR)** If subjective cognitive decline or decline in functional ability is perceived by the clinician.
**(CR)** If reduced cognition is demonstrated on a brief global cognitive screen.
**(SR)** If behavioural and/or psychological symptoms are present.
**(CR)** When functional neurosurgery (e.g., DBS, Gamma knife) is being considered.
**(CR)** When there is diagnostic uncertainty or for the purpose of differential diagnosis.
**(CR)** If capacity assessment (e.g., for guardianship) is required.
**(CR)** If the person living with PD requests a cognitive evaluation.

LR *3.3.2 Any clinician, including allied health professionals (e.g., occupational therapist (OT), physiotherapist or speech pathologist), should be able to make a referral to a clinical neuropsychologist if there are concerns about the presence of cognitive changes.*

3.3.2 Why is this important?
Allied health professionals may have regular contact with people living with PD and greater opportunities to detect cognitive decline over time.

Comprehensive neuropsychological assessment toolkit

CR *3.3.3 Due to its relatively greater sensitivity to mild cognitive impairments in PD, the Montreal Cognitive Assessment (MoCA) is the preferred brief test of global cognition in PD.*

CR *3.3.4 Consider self-report tools, informant interview, observational evaluation and formal testing for assessment of Activities of Daily Living (ADL) to identify functional impairment due to cognitive impairment rather than motor impairment.*

CR

3.3.5 Neuropsychological test batteries to identify cognitive disorders in PD should be designed to minimise duration of the test battery to prevent fatigue and to mitigate influence of motor symptomatology on the test results.

Preparing for a comprehensive neuropsychological assessment

CR

3.3.6 People living with PD and their care partners should receive verbal and written information regarding the context and purpose of their comprehensive neuropsychological assessment to prepare for the appointment.

CR

3.3.7 Referral to an experienced psychologist should be considered if people living with PD and/or their care partner express emotional distress during the waiting time for the comprehensive neuropsychological assessment and require support.

LR

3.3.8 Clinicians should clarify contact details of other medical and allied health professionals of people living with PD so that they may be informed of the outcomes of comprehensive neuropsychological assessments.

LR

3.3.9Clinicians should discuss whether people living with PD want to know the assessment outcome or whether this information should only be communicated with their care partners or support people.

LR

3.3.10 People living with PD should be made aware of the purpose and potential outcomes of comprehensive neuropsychological assessments.

3.3.10 Why is this important?
People living with PD considering a comprehensive neuropsychological assessment should be provided with sufficient information to make an informed decision. Potentially negative outcomes of comprehensive neuropsychological assessments may include a diagnosis of mild cognitive impairment or dementia, loss of legal or financial capacity, and loss of medical fitness to drive. Potentially positive outcomes of comprehensive neuropsychological assessments may include enhanced care, treatment of cognitive symptoms, future planning, and improved interpersonal relationships.

Assessment procedures

LR

3.3.11 If a clinician asks a care partner or a support person about the current cognitive function of a person living with PD, the person being assessed should be asked whether they want to be present during that conversation or not.

3.3.11 Why is this important?
The person living with PD should be given the opportunity to decide whether they wish to be present during discussions, upholding the principles of autonomy, privacy and dignity. Their decision should also be respected if they choose not to be present.

CR

3.3.12 Efforts to stabilise affective, mood, or psychiatric conditions should be made prior to cognitive evaluation, as this may influence test administration and results.

CR

3.3.13 People living with PD should be tested in the ON-state (i.e., when parkinsonian symptoms are controlled), unless specific OFF-state testing is required.

CR

3.3.14 Where possible, consider aligning comprehensive neuropsychological assessment with antiparkinsonian medication regimes to ensure that evaluations are conducted during the "ON-state".

3.3.14 Why is this important?
Fluctuations in motor (e.g., tremor) and non-motor (e.g., anxiety) symptoms may influence performance on neuropsychological assessments. Conducting all cognitive evaluations in the "ON-state" may minimise this impact.

PP

3.3.15 Telehealth neuropsychological assessments should be made available to people living with PD who cannot access neuropsychology services in person.

3.3.15 Why is this important?
Diagnosis of cognitive disorders is not possible without cognitive evaluation. While evidence for tele-neuropsychology in PD is in its infancy, every effort should be made to service people who are unable to access in person assessments (e.g., those in rural settings, with mobility issues).

CR

3.3.16 People living with PD who are from Culturally and Linguistically Diverse (CALD) backgrounds should have access to interpreters and/or translated versions of neuropsychological assessments where possible to facilitate assessment and minimise cultural bias.

Feedback sessions

SR

3.3.17 A feedback session should be offered to people living with PD and their care partners within one month of their comprehensive neuropsychological assessment to receive any formal diagnoses and discuss a care plan.

SR

3.3.18 Feedback of comprehensive neuropsychological assessment results should be delivered with sensitivity and appropriate consent.

3.4 Post-diagnostic care

Post-diagnostic care for people living with PD and cognitive impairment include treatment (pharmacological and non-pharmacological), support, and care planning. The following recommendations pertain to best practices in the post-diagnostic care of people living with PD.

Pharmacological treatment

CR

3.4.1 The following drugs could be considered in the treatment of dementia in PD: [30]

**First Line**
Rivastigmine
Donepezil

**Second Line**
Galantamine
NMDA antagonists (Memantine)

3.4.1 Practice point:
Consider transdermal application of rivastigmine in preference to oral administration for reduced gastrointestinal side effects and improved tolerability.

CR

3.4.2 Cautious deprescribing of medications should be considered after diagnosis of any cognitive disorder in PD, including:

Anticholinergics (e.g., tolterodine, oxybutynin, tricyclic antidepressants)
Benzodiazepines (e.g., alprazolam, diazepam)
Antipsychotics with high affinity for D2 receptors (e.g., haloperidol, risperidone)

CR

3.4.3 For people experiencing cognitive disorders in PD, antipsychotics (e.g., clozapine, quetiapine) should be prescribed at the lowest dose for the shortest possible time, reassessing their need every 12 weeks.

CR

3.4.4 Polypharmacy should be minimised where possible for people experiencing cognitive disorders in PD.

3.4.4 Why is this important?
Reducing polypharmacy in people with cognitive disorders will improve adherence to medication and reduce the risk of cognitive side effects from various medications.

Non-pharmacological treatment

CR

3.4.5 The following non-pharmacological treatments for people living with PD who receive any cognitive diagnosis should be considered: [30]

Goal-focused rehabilitation (e.g., assistive technology, home modifications)
Cognitive rehabilitation (cognitive exercises)
Cognitive rehabilitation (computerised cognitive training)
Activities of Daily Living (ADL) training/support
Memory strategy training
Dance exercise
Music contingent gait training
Tai chi

Lifestyle modifications

SR

3.4.6 The following lifestyle modifications should be discussed with people living with PD who receive any cognitive diagnosis and their care partners:

Community mobility and home safety strategies
Falls prevention strategies
Physical activity
Cognitive stimulation (e.g., puzzles, crosswords)
Social engagement
Mediterranean diet

CR

3.4.7 People living with PD with a cognitive diagnosis should be advised to:

Take their time while completing tasks
Let their support network know if they are having trouble
Seek help if depressed or anxious
Develop a highly structured daily routine to follow
Develop cognitive coping strategies (e.g., for attention, memory) with occupational therapists or psychologists

Care services

SR

3.4.8 People living with PD should receive advice on or referral to the following healthcare services after receiving any cognitive diagnosis:

Dementia Australia
Dietician
Occupational therapist
Old age psychiatrist
Physiotherapist
Psychoeducation
Psychologist
Social worker
Speech therapist
Exercise physiologist

PP

3.4.9 Healthcare services for cognitive impairment (e.g., psychoeducation, allied health) should also be offered to people living with PD experiencing subjective cognitive decline.

3.4.9 Practice point:
These services may not always be available for people experiencing subjective cognitive decline

Post-diagnostic care plan

CR

3.4.10 A detailed post-diagnostic care plan should be developed in partnership with people living with PD and their care partners soon after a cognitive diagnosis and monitored during follow up appointments.

SR

3.4.11 The post-diagnostic care plan should be shared with people living with PD and their care partners and include links to online resources and contact details for relevant support services (e.g., psychoeducation, support groups, counselling, legal aid).

CR

3.4.12 The primary care physician (e.g., GP) should be involved in the implementation of the post-diagnostic care plan where possible.

Important discussions

LR

3.4.13 After a diagnosis of mild cognitive impairment or dementia, clinicians should be mindful to speak to both people living with PD and their care partners in consults and not exclude either person.

CR

*3.4.14 Advance care planning / directives and estate planning should be discussed with people living with PD and their care partners soon after diagnosis of PD and reconsidered **soon after diagnosis** of any cognitive disorder.*

3.4.14 Why is this important?
End of life planning should be discussed as soon as possible without causing major distress to the patient. In a sensitive yet timely manner, endeavour to discuss these topics with the patient and their care partner before capacity is lost.

CR

*3.4.15 Sensitive, tailored discussions regarding fitness to drive should be considered for people living with PD and their care partners soon after diagnosis of PD and again **soon after diagnosis** of any cognitive disorder.*

CR

*3.4.16 Supports and adjustments for people currently working should be discussed with people living with PD **soon after diagnosis** of any cognitive disorder.*

CR

*3.4.17 Community mobility monitoring/planning should be discussed with people living with PD and their care partners **soon after diagnosis** of any cognitive disorder to provide adjustment support for transitions with mobility.*

CR

3.4.18 People experiencing cognitive disorders in PD and their care partners should be made aware of any local clinical trials and/or relevant local support services (e.g., dementia support groups or support groups for care partners) of potential relevance.

4 Neuropsychological Toolkit

The PDCogniCare Standardised Neuropsychological Toolkit ('the Toolkit') provides recommendations for neuropsychological tools to identify and diagnose cognitive disorders in PD. These recommendations may be adopted by any clinicians involved in the administration of cognitive evaluation to people living with PD. The Toolkit comprises a cognitive test battery endorsed by the PDCogniCare clinician and research expert Delphi panel. All tests within the battery have demonstrated predictive utility for identifying cognitive decline in PD.[31]

4.1 Recommended measures

The following measures are recommended for cognitive evaluations in PD due to their sensitivity to cognitive decline in PD, reasonable duration, availability of alternative forms, and limited interference of motor symptoms:

Cognitive Domain	Measure
**Verbal memory**[1]	Rey Auditory Verbal Learning Test
	California Verbal Learning Test
	Hopkins Verbal Learning Test
**Visual memory**[1]	Rey-Osterrieth Complex Figure Test
	Brief Visuospatial Memory Test
**Executive function**	Trail-Making Test [B]
	STROOP Word-Colour scale
**Attention/Processing speed**	Symbol Digit Modalities Test
	Trail-Making Test [A]
	STROOP Word or Colour scales
**Visuospatial function**	Pentagon copying
**Fluency**	Category fluency
**Global cognition**	Montreal Cognitive Assessment

[1]Select one test

4.2 Intended use

The Toolkit is designed to inform neuropsychological test choice for clinicians investigating cognitive impairment in PD, with the intention to standardise diagnostic processes for cognitive disorders in PD. Measures of the Toolkit were chosen specifically for their predictive utility in PD to develop a test battery uniquely sensitive to cognitive decline in PD. Measures from the Toolkit may be selected to form an abridged test battery, although it is recommended that the measures are administered as a whole test battery. Currently, gold standard criteria for the diagnosis of cognitive

disorders in PD include the Movement Disorder Society (MDS) criteria for PD-MCI or PDD,[18, 22] or Diagnostic Statistical Manual (DSM-5-TR) criteria for minor or major neurocognitive disorder due to PD.[23] While the present guidelines recommend application of the DSM-5-TR criteria for neurocognitive disorder due to PD, the Toolkit may be used to align with either criteria. Optimisation of the Toolkit is ongoing, with the aim of validating the diagnostic accuracy of the Toolkit, operationalising standardised parameters for identifying 'objective' impairment, and exploring cost-effective applications of the Toolkit in both face-to-face and telehealth modalities.

5 Dissemination

5.1 Implementation

Implementing the PDCogniCare best practice guidelines in movement disorders clinics or other clinical settings where PD patients are seen will involve a structured approach to ensure accurate diagnosis and effective management of cognitive disorders. All clinical staff involved in PD care within a given setting should be informed of the guidelines and how to access them. Ideally, staff will receive training on key elements of the guidelines related to their practice. For example, movement disorders nurses involved in conducting brief cognitive screens and referring patients for comprehensive neuropsychological assessment will receive specific advice on these procedures in accordance with the guidelines. Training of multidisciplinary staff members involved in PD care will facilitate the establishment of clear referral pathways and care coordination both within and across clinical settings.

5.2 Barriers and Facilitators

Information from pre-implementation stakeholder interviews conducted for the PDCogniCare provided insight into barriers and facilitators for the best practice guidelines for cognitive disorders in PD. An important barrier included limitations in clinic resources (e.g., staff, time, funding), which can hinder the ability to refer for or conduct comprehensive neuropsychological assessments and follow-ups. This is particularly relevant for busy or overburdened movement disorders clinics. Limitations in neuropsychology services was also identified as another potential barrier, particularly in regional and rural settings. In these cases, it may be difficult to implement some of the recommendations within the present guidelines and provide training to staff on appropriate use of the guidelines. Another potential barrier to guideline implementation was attitudes toward cognitive evaluation, such as the perceived benefits of evaluations and how they inform management of patients. Similarly, the influence of neuropsychologist preference in the selection of cognitive measures may be another barrier that may reduce openness to adopting the guidelines and standardised toolkit.

Conversely, facilitators to implementation of the guidelines include the PDCogniCare Training Package, which provides comprehensive training for the guidelines. The integration of the guidelines into the electronic PDCogniCare platform may also promote uptake of the guidelines. The collaborative involvement of various stakeholders in the development and adaptation of the guidelines to fit the Australian context also increases likelihood of its acceptance and implementation. Specifically, various PD clinicians across Australia contributed to the Delphi panel and many people with lived experience were involved in the development of the recommendations, which may promote awareness of the guidelines among these communities.

6 Future directions

Recent advancements in biomarker discovery, AI-driven diagnostics, and disease-modifying therapeutics for neurodegenerative disease provide insight into the potential future of cognitive research in PD. Biomarkers aims to enhance early diagnosis, prognosis, and treatment of cognitive disorders in PD. Emerging literature exploring cerebrospinal fluid, imaging, and blood biomarkers show promise for identifying people living with PD at risk of developing dementia.[34] AI-driven models have also demonstrated potential to revolutionise evaluation and management of cognitive disorders through the integration of multimodal data to improve diagnostic accuracy, predict cognitive decline, identify subtypes of cognitive impairment, and personalise and optimise treatment interventions.[35, 36] Finally, recent scientific breakthroughs in disease-modifying therapeutics instil hope for future treatment of cognitive impairment in PD, with monoclonal antibodies in the treatment of Alzheimer's disease and gene therapy targeting PD motor symptoms paving the way.[37, 38] As these technologies evolve, so too will the best practice guidelines in an effort to maintain current, evidence-based practices and a high quality of care for people living with PD.

Beyond advances in research, the PDCogniCare project will continue to progress the implementation and evaluation of standardised best practice guidelines to determine their cost-effectiveness and scalability in Australian clinical settings. International collaboration will also be critical to promote the standardisation of cognitive evaluation methods and quality of care standards across PD populations globally. Regular updates to the guidelines will ensure they remain evidence-based and adaptable, with the ultimate goal of improving quality of life and health outcomes for people experiencing cognitive disorders in PD.

7 References

1. Australian Institute of Health and Welfare. Dementia in Australia. Canberra: AIHW; 2024.
2. Dementia Australia. The Economic Cost of Dementia in Australia 2016–2056. Dementia Australia; 2017.
3. Feigin VL, Nichols E, Alam T, Bannick MS, Beghi E, Blake N, et al. Global, regional, and national burden of neurological disorders, 1990-2016: a systematic analysis for the Global Burden of Disease Study 2016. Lancet Neurol. 2019;18(5):459-80. DOI:10.1016/S1474-4422(18)30499-X
4. Hely MA, Reid WG, Adena MA, Halliday GM, Morris JG. The Sydney multicenter study of Parkinson's disease: the inevitability of dementia at 20 years. Mov Disord. 2008;23(6):837-44. DOI:10.1002/mds.21956
5. Li Y, McLernon DJ, Counsell CE, Macleod AD. Incidence and risk factors of institutionalisation in Parkinson's disease and atypical parkinsonism. 2024;118:105928. DOI:10.1016/j.parkreldis.2023.105928
6. Leroi I, McDonald K, Pantula H, Harbishettar V. Cognitive impairment in Parkinson disease: impact on quality of life, disability, and caregiver burden. J Geriatr Psychiatry Neurol. 2012;25(4):208-14. DOI:10.1177/0891988712464823
7. Vossius C, Larsen JP, Janvin C, Aarsland D. The economic impact of cognitive impairment in Parkinson's disease. Mov Disord. 2011;26(8):1541-4. DOI:10.1002/mds.23661
8. Deloitte Access Economics Report: Living with Parkinson's Disease. 2015.
9. Aarsland D, Batzu L, Halliday GM, Geurtsen GJ, Ballard C, Ray Chaudhuri K, et al. Parkinson disease-associated cognitive impairment. Nat Rev Dis Primers. 2021;7(1):47. DOI:10.1038/s41572-021-00280-3
10. Pourzinal D, Yang JHJ, Bakker A, McMahon KL, Byrne GJ, Pontone GM, et al. Hippocampal correlates of episodic memory in Parkinson's disease: A systematic review of magnetic resonance imaging studies. J Neurosci Res. 2021;99(9):2097-116. DOI:10.1002/jnr.24863
11. Pourzinal D, Yang J, McMahon KL, Copland DA, Mitchell L, O'Sullivan JD, et al. Hippocampal resting-state connectivity is associated with posterior-cortical cognitive impairment in Parkinson's disease. Brain Behav. 2024;14(3):e3454. DOI:10.1002/brb3.3454
12. Yang J, McMahon KL, Copland DA, Pourzinal D, Byrne GJ, Angwin AJ, et al. Semantic fluency deficits and associated brain activity in Parkinson's disease with mild cognitive impairment. Brain Imaging Behav. 2022;16(6):2445-56. DOI:10.1007/s11682-022-00698-7
13. Pourzinal D, Lawson RA, Yarnall AJ, Williams-Gray CH, Barker RA, Yang J, et al. Profiling people with Parkinson's disease at risk of cognitive decline: Insights from PPMI and ICICLE-PD data. Alz Demen DADM. 2024;16(3):e12625. DOI:10.1002/dad2.12625
14. Lawson RA, Yarnall AJ, Duncan GW, Khoo TK, Breen DP, Barker RA, et al. Quality of Life and Mild Cognitive Impairment in Early Parkinson’s Disease: Does Subtype Matter? J Parkinsons Dis. 2014;4:331-6. DOI:10.3233/JPD-140390

15. Aarsland D, Bronnick K, Williams-Gray C, Weintraub D, Marder K, Kulisevsky J, et al. Mild cognitive impairment in Parkinson disease: a multicenter pooled analysis. Neurol. 2010;75(12):1062-9. DOI:10.1212/WNL.0b013e3181f39d0e

16. Yang J, Pourzinal D, Byrne GJ, McMahon KL, Copland DA, O'Sullivan JD, et al. Global assessment, cognitive profile, and characteristics of mild cognitive impairment in Parkinson's disease. Int J Geriatr Psychiatry. 2023;38(6):e5955. DOI:10.1002/gps.5955

17. Lawson RA, Yarnall AJ, Duncan GW, Breen DP, Khoo TK, Williams-Gray CH, et al. Stability of mild cognitive impairment in newly diagnosed Parkinson's disease. J Neurol Neurosurg Psychiatry. 2017;88(8):648-52. DOI:10.1136/jnnp-2016-315099

18. Litvan I, Goldman JG, Tröster AI, Schmand BA, Weintraub D, Petersen RC, et al. Diagnostic criteria for mild cognitive impairment in Parkinson's disease: Movement Disorder Society Task Force guidelines. Mov Disord. 2012;27(3):349-56. DOI:10.1002/mds.24893

19. Pourzinal D, Yang J, Lawson RA, McMahon KL, Byrne GJ, Dissanayaka NN. Systematic review of data-driven cognitive subtypes in Parkinson disease. Eur J Neurol. 2022;29(11):3395-417. DOI:10.1111/ene.15481

20. Wood KL, Myall DJ, Livingston L, Melzer TR, Pitcher TL, MacAskill MR, et al. Different PD-MCI criteria and risk of dementia in Parkinson's disease: 4-year longitudinal study. NPJ Parkinsons Dis. 2016;2:15027. DOI:10.1038/npjparkd.2015.27

21. Nicoletti A, Luca A, Baschi R, Cicero CE, Mostile G, Davì M, et al. Incidence of Mild Cognitive Impairment and Dementia in Parkinson's Disease: The Parkinson's Disease Cognitive Impairment Study. Front Aging Neurosci. 2019;11:21. DOI:10.3389/fnagi.2019.00021

22. Goetz CG, Emre M, Dubois B. Parkinson's disease dementia: definitions, guidelines, and research perspectives in diagnosis. Ann Neurol. 2008;64 Suppl 2:S81-92. DOI:10.1002/ana.21455

23. American Psychiatric Association. Diagnostic and Statistical Manual of Mental Disorders: American Psychiatric Association; 2013.

24. Severiano e Sousa C, Alarcão J, Pavão Martins I, Ferreira JJ. Frequency of dementia in Parkinson's disease: A systematic review and meta-analysis. J Neurological Soc. 2022;432:120077. DOI:10.1016/j.jns.2021.120077

25. Severiano e Sousa C, Fabbri M, Godinho C, Simões R, Chendo I, Coelho M, et al. Clinical Diagnostic Criteria Have a High Impact on the Frequency of Dementia in Late-Stage Parkinson's Disease. Front Neurol. 2021;12. DOI:10.3389/fneur.2021.652424

26. Goldman JG, Vernaleo BA, Camicioli R, Dahodwala N, Dobkin RD, Ellis T, et al. Cognitive impairment in Parkinson's disease: a report from a multidisciplinary symposium on unmet needs and future directions to maintain cognitive health. NPJ Parkinsons Dis. 2018;4:19. DOI:10.1038/s41531-018-0055-3

27. Carney SF. Nobody Really Knows Us. UK: Parkinson's UK; 2021.

28. Pigott JS, Davies N, Chesterman E, Read J, Nimmons D, Walters K, et al. Delivering Optimal Care to People with Cognitive Impairment in Parkinson's Disease: A Qualitative Study of Patient, Caregiver, and Professional Perspectives. Parkinsons Dis. 2023;2023:9732217. DOI:10.1155/2023/9732217

29. Postuma RB, Berg D, Stern M, Poewe W, Olanow CW, Oertel W, et al. Abolishing the 1-year rule: How much evidence will be enough? Mov Disord. 2016;31(11):1623-7. DOI:10.1002/mds.26796
30. Pourzinal D, Elgey C, Bailey DX, Yang J, Lehn A, Tinson H, et al. Diagnosis, evaluation & management of cognitive disorders in Parkinson's disease: A systematic review. Int Psychogeriatrics. 2025:100081. DOI:10.1016/j.inpsyc.2025.100081
31. Pourzinal D, King J, Sivakumaran K, Yang J, McCann E, Mitchell LK, et al. Evaluating evidence for a neuropsychological toolkit to predict cognitive decline in PD: A systematic review. 2025:1-28. DOI:10.1080/13854046.2025.2511966
32. Pourzinal D, Brooks D, Sriram D, Mitchell LK, Pachana NA, Shrubsole K, et al. Diagnosis, evaluation and management of cognitive disorders in Parkinson's disease: Consensus recommendations from a modified Delphi process. 2025.
33. Sriram D, Pourzinal D, Bailey DX, Brooks D, Shrubsole K, Yang J, et al. Recommendations to improve healthcare service provision for cognitive impairment in Parkinson's disease: a mixed methods study of the lived experience expert perspective. 2025;In press.
34. Mantovani E, Martini A, Dinoto A, Zucchella C, Ferrari S, Mariotto S, et al. Biomarkers for cognitive impairment in alpha-synucleinopathies: an overview of systematic reviews and meta-analyses. NPJ Parkinsons Dis. 2024;10(1):211. DOI:10.1038/s41531-024-00823-x
35. Dennis A-GP, Strafella AP. The identification of cognitive impairment in Parkinson's disease using biofluids, neuroimaging, and artificial intelligence. Front Neurosci. 2024;18. DOI:10.3389/fnins.2024.1446878
36. Ortelli P, Ferrazzoli D, Versace V, Cian V, Zarucchi M, Gusmeroli A, et al. Optimization of cognitive assessment in Parkinsonisms by applying artificial intelligence to a comprehensive screening test. NPJ Parkinsons Dis. 2022;8(1):42. DOI:10.1038/s41531-022-00304-z
37. Currie AD, Wong JK, Okun MS. A review of temporal interference, nanoparticles, ultrasound, gene therapy, and designer receptors for Parkinson disease. NPJ Parkinsons Dis. 2024;10(1):195. DOI:10.1038/s41531-024-00804-0
38. Mahboob A, Ali H, AlNaimi A, Yousef M, Rob M, Al-Muhannadi NA, et al. Immunotherapy for Parkinson's Disease and Alzheimer's Disease: A Promising Disease-Modifying Therapy. Cells. 2024;13(18). DOI:10.3390/cells13181527
